# Effects of Calcium Silicate Slag on Hydration of Cementitious Pastes

**DOI:** 10.3390/ma12193094

**Published:** 2019-09-23

**Authors:** Ju Zhang, Changwang Yan, Pucun Bai, Xiaoxiao Wang, Shuguang Liu, Zhigang Liu

**Affiliations:** 1 School of Materials Science and Engineering, Inner Mongolia University of Technology, Hohhot 010051, China; zj970741@126.com (J.Z.); wxiaoxiao.good@163.com (X.W.); liusg6011@126.com (S.L.); 2 School of Mining and Technology, Inner Mongolia University of Technology, Hohhot 010051, China; 13039508643@163.com

**Keywords:** calcium silicate slag, cementitious pastes, chemically combined water, beta-dicalcium silicate, flexural strength, pore structure, micromorphology

## Abstract

Calcium silicate slag (CSS) is waste slag and it contains a large amount of beta-dicalcium silicate. This study is mainly focused on the effect of CSS on the hydration of cementitious pastes. CSS was used to partly replace cement, and composite pastes containing CSS and cement were prepared. The mineral composition and particle size distribution of CSS were characterized. The chemically combined water of the paste sample was measured at a given test age. Based on the value of chemically combined water, the hydration degree and the hydration rate of composite pastes were analyzed. The flexural strength of the samples was established. The pore structure and micromorphology of the sample were also observed. The results indicate the chemically combined water decreased, the hydration degree decreased, the hydration rate declined, and the spherical micromorphology of the calcium silicate hydrate gel was reduced after more cement was replaced by CSS in the composite pastes. Besides, the amount of pores increased, its size was bigger, and air content in the pore was higher. However, flexural strength was lower. CSS has a significant impact on the hydration of cementitious pastes, and it is thus suitable to regulate hydration.

## 1. Introduction

Chemically combined water is a part of the hydration products of cement [1,2]. A faster hydration rate can generate more hydration products and more chemically combined water [3]. However, the hydration rate of cement needs to be decreased in large concrete construction projects, such as dams [4], foundations [5], and piers [6]. A slower hydration rate or less chemically combined water content for cementitious materials can reduce hydration heat and help to control cracks during construction. 

Some measures have been adopted to decrease chemically combined water and slow the hydration rate. The chemically combined water of blends has been reduced after cement has been partly replaced with electric arc furnace slag [7,8]. Saraya [9] conducted a comparative study and found that the hydration rate of cement was much slower after blast furnace slag was blended. The least amount of chemically combined water occurred for cement pastes with basalt pozzolanic, up to 90 days. Cement pastes with limestone contained a lower chemically combined water content than those with blast furnace slag, silica fume and ordinary Portland cement, but they contained a higher than one with basalt pozzolanic. In order to lower the amount of chemically combined water in Portland cement, Rahhal et al. used silica fume, diatomite, quartz, metakaolins, and gypsum were used [10,11]. Their study results showed the amounts of chemically combined water occurred most for the cement with silica fume and diatomite, and these amounts were the least for the cement with quartz because of its insignificant pozzolanic activity. Zhang et al. [12] used large-volume fly ash to decrease the chemically combined water of cementitious materials. The experimental results showed that the amount of chemically combined water was lower in the cement sample with large-volume fly ash than that in the pure cement sample. The same results were found by Berry [13] and Lam [14]. By contrast, some materials could improve the nucleation of C–H and accelerate hydration, and chemically combined water of ordinary Portland cement content increased with the increase of the material contents such as nano-clay [15], nano-silica [16] and limestone [17].

Calcium silicate slag (CSS) is waste slag from the extraction of alumina from fly ash [18,19]. After a ton of alumina is extracted, approximately 2.2 tons of CSS waste slag is produced [20]. Hence, CSS maybe covers a large area of land, and its small particles may pollute the environment. After a series of tests [21,22], it was found that CSS contained a large amount of beta-dicalcium silicate (β-C_2_S). In the concrete mixture, β-C_2_S can slowly hydrate [23]. The chemically combined water contents increased at early ages of β-C_2_S hydration up to 14 days [24]. Thus, it is possible that CSS could be used to regulate the hydration rate and chemically combined water of cement. 

In this paper, CSS was partly used to replace cement, and composite mixtures containing CSS and cement were prepared. The characteristics of CSS were analyzed by X-ray diffraction (XRD) patterns. The chemically combined water of the sample was measured at a certain test age. According to the value of chemically combined water, the hydration degree and hydration rate of composite mixtures were analyzed. Besides, the micromorphology of the sample was observed by scanning electron microscopy.

## 2. Experimental Procedure 

### 2.1. Sample Preparation

The CSS samples in this study were collected from Datang International Renewable Resources Development Co. Ltd. in Inner Mongolia, China. Their main chemical components are listed in Table 1. The CSS samples were processed by ball milling for 30 min and dried for 24 h at 105 °C in an oven box. The size distribution of the CSS was measured using a WJL-606 laser particle sizer, and 90% of the total particle sizes were less than 18 μm. Ordinary Portland cement, type P.O42.5, was used in this study and was provided by the Jidong cement plant (Huhhot, China).

The samples were made of CSS and cement. The mix proportions are shown in Table 2. Three samples were prepared per composition. The CSS contents by mass in the paste samples were 0, 10%, 20%, 30%, 40%, 50% and 60%. The water-to-binder (W/B) ratios were 0.35, 0.40 and 0.45. The sample was molded, demolded, and cured in a standard curing box with a temperature of 20 ± 1 °C and a relative humidity of 95%. The sample was a cube, and the length of its sides was 20 mm. The test ages were 1, 3, 7, 14, 28, 56 and 90 d. At each test age, the cubic sample was immersed in absolute ethanol for 3 d in order to stop hydration, and then 3 mm was removed from the surface of the sample. The remainder of the sample was ground into a powder by a crucible. The powder was separated by a square mesh sieve with 200 orders, and the sieve residue was less than 5%. The treated sample was sealed in a self-sealing bag for further measurement.

### 2.2. Methods

Chemically combined water was measured in this study. One gram of the treated sample was weighed and placed in a drying box. The drying temperature was kept at 80 °C until the weight of the sample was constant (*m*_1_). Then, the sample was quickly put into a muffle furnace again. The temperature of the muffle furnace rose to 950 °C continuously at a rate of 10 °C/min and was kept constant. The sample was weighed every 10 min. When the weight difference was less than 5%, it was considered to have reached a constant value (*m*_2_). Each sample was measured three times using the same method. Chemically combined water W*_t_* at the age of t could be calculated by Equations (1) and (2).

(1) $$W_{t}=\frac{m_{1}-m_{2}}{m_{2}}-\frac{L}{1-L}$$

(2) $$L=L_{CS}W_{CS}+L_{C}W_{C}$$

where *L* is the loss on ignition (*LOI*); *L*_CS_ and *L*_C_ are the LOI of the CSS and cement, respectively; and *W*_CS_ and *W*_C_ are the mass percentage of the CSS and cement in the mix proportion, respectively.

According to *W_t_*, the hydration degree *d*(t) and hydration rate *r*(t) of the paste sample can be obtained from by Equations (3) and (4).

(3) $$d(t)=W_{t}/W_{\infty }$$

(4) $$r(t)=\frac{W_{t}-W_{t-1}}{A_{t}-A_{t-1}}$$

where *W*_∞_ and *W_t_* are chemically combined water at the age of 90th day and *t*, and *A_t_* and *A_t−1_* are the adjacent measure ages.

A Panalytical X’Pert X-ray diffractometer (Almelo, The Netherlands) was used to identify the characteristics of CSS. The accelerating voltage was 40 kV, and the current was 40 mA. The treated sample was examined between 5° and 70° 2θ at scanning rate of 5° 2θ per minute. The diffractograms were obtained with X Pert Highscore software (X Pert Highscore Plus 2.0, Phylips, Eindhoven, The Netherlands), and the mineral composition of CSS was identified. 

A rapid Air 457 concrete pore structure analyzer (Shanghai, China) was used to measure the pore structure and specific surface of the cubic sample. The working procedures of cutting, grinding, polishing, cleaning and drying at 105 °C were adopted in turn. Then, the test surface was blackened with stamp pad, and zinc oxide thick paint was applied on the surface. 

The flexural strength was tested by a DYH-300B automatic cement pressure testing machine (Shanghai, China) at the age of 90 days. The loading rate was 50 ± 10 N/s. The sample was prismatic, and its sizes were 40, 40 and 160 mm.

The morphology of the paste sample at a certain age was observed using scanning electron microscopy (SEM) on a Hitachi S-4800 instrument (Tokyo, Japan). The accelerating voltage was 20 kV, the decelerating voltage was 0 volts, and the emission current was 10 μA. All the samples were blocks with a size of 2 mm and were treated by spray-gold using E-1010 ion sputtering equipment (Hitachi, Tokyo, Japan).

## 3. Results and Analysis

### 3.1. Characteristics of CSS

**Figure 1:**
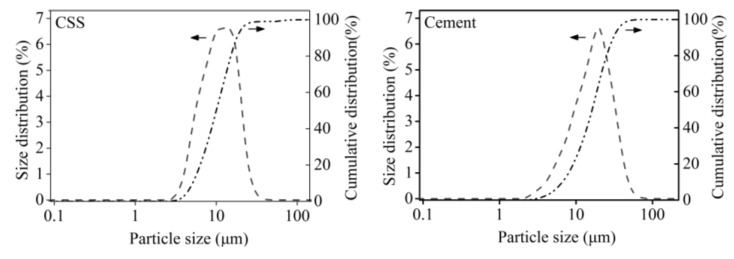
Particle size distribution of CSS and cement.

CSS is the waste slag from the extraction of alumina from fly ash. Its main chemical components are listed in Table 1. The type and content of chemical components were similar when CSS was compared with cement. The particle size distributions of CSS and cement are shown in Figure 1. It could be found that the size distribution and cumulative distribution were approximately the same. Their maximum particle sizes were equivalent. Based on the results of main chemical components and particle size distribution, it can be said that CSS can be activated by mechanical ball-milling.

**Figure 2:**
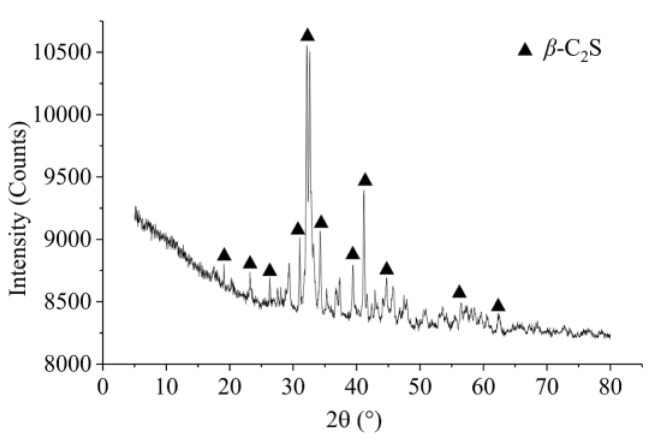
XRD pattern of CSS.

The mineral composition of CSS was identified by XRD, and its XRD pattern is shown in Figure 2. It can be seen in the pattern that beta-dicalcium silicate (β-C_2_S) was the main mineral component, and the proportion was approximately 90%. Therefore, the CSS had hydration characteristics similar to those of cement, and its hydration was slower than that of cement. After the cement was partially replaced by CSS, the chemically combined water of cement could be regulated.

### 3.2. Quantitative Analysis of Chemically Combined Water

**Figure 3:**
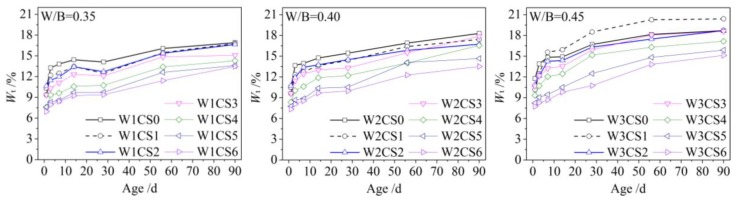
Influence of CSS contents on chemically combined water.

The value of *W_t_* was obtained according to Equations (1) and (2), as shown in Figure 3. The value of *W_t_* increased with increasing age. For the samples containing a small amount of CSS, *W_t_* increased significantly within 14 d of age. More chemically combined water could be obtained easily in the short term, and the hydration degree exceeded 75% because the hydration rate of the Portland cement was fast. This phenomenon was the same as for straight cement, such as samples W1CS0, W2CS0 and W3CS0. 

After more CSS was added into the sample, there was less chemically combined water because the mineral composition of CSS was mainly β-2CaO·SiO_2_, and its hydration reaction was slower. After 14 d of age, all *W_t_*–age curves were gradual, and the increase in W*_t_* was small. The hydration rate of all samples developed smoothly with age, and the increase in the hydration degree slowed because most cement in the samples was consumed by the hydration reaction. The hydration of the CSS began to play a major role in this stage, and more time was needed to reach a high hydration degree. At the age of 90 d, the hydration of the samples was not yet completed.

Furthermore, *W_t_* slightly increased with an increase in the W/B ratio for the sample containing the same CSS content, as shown in Figure 3. The reason may be that the water increased for the sample with the increased W/B ratio, and it could promote scatter the hydration reactants and products thoroughly. In this way, additional interfaces and space were provided in the paste sample to hydration activity. Consequently, chemically combined water was abundant in the sample.

### 3.3. Hydration Degree

**Figure 4:**
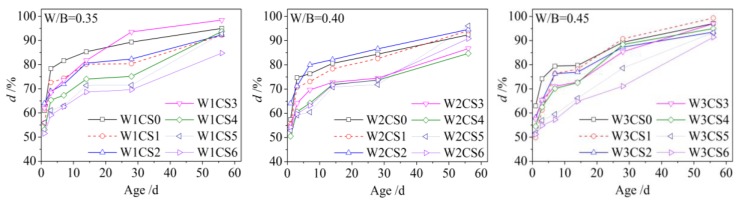
Hydration degree of the sample at a different age.

According to Equation (3), hydration degree (d) was obtained, as shown in Figure 4. For all samples, hydration degree increased with increases of age. Two evident stages appeared in the *d*–age curves. The age of 14 days was the demarcation point. The curves were steeper, and the increase was faster in the first stage than in the second stage. Because there was less CSS content, the samples containing more cement rapidly hydrated, and the increase of d was faster in the first stage. Hydration degree reached above 75% within 14 days. When more CSS was added into the sample, the increase of d became slower. When comparing the sample with less CSS with more CSS, it could be seen that the maximum of slow amplitude was about 20%. A similar thing happened to chemically combined water of the sample. The change trend of d had a good agreement with one of *W*_t_.

After the age of 14, the increase of d slowed down because most cement in the samples as consumed by the hydration reaction. The slower hydration of CSS began to play a major role in the second stage. The increment of chemically combined water reduced, and more time was needed to reach a higher hydration degree. At the age of 56, the hydration of the samples was not yet completed, and the hydration degree was approximately 90%. 

It can be seen from Figure 4 that the W/B ratio had little effect on hydration degree. In the sample, *W_t_* came mainly from the cement because of the lower hydration action of CSS. As shown in Figure 3, chemically combined water hardly changed with an increase of W/B for the sample without CSS. The W/B had little impact on the hydration of cement. Additionally, above 80% of chemically combined water could be obtained for the sample without CSS before the age of 14. The difference between the *W_t_* and *W*_∞_ was small in Equation (3). Thus, the hydration degree was almost the same for the samples with different W/B ratios.

### 3.4. Hydration Rate 

**Figure 5:**
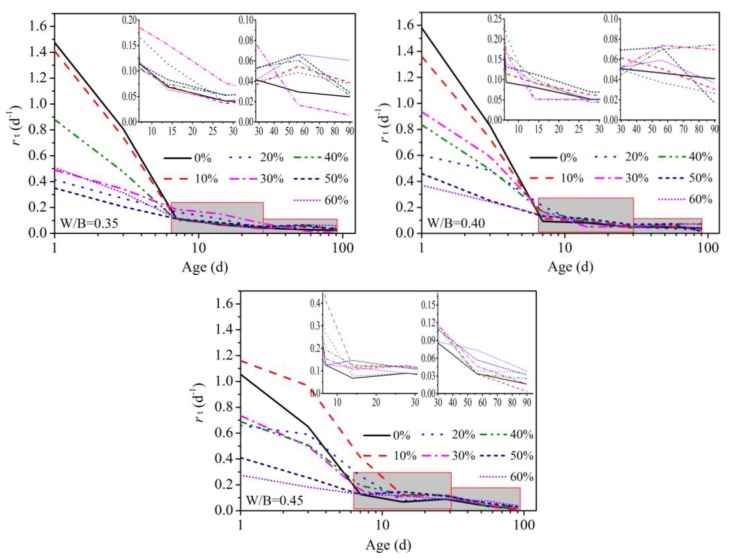
Hydration rate of the sample.

According to Equation (4), hydration rate (*r*) of the samples was obtained, and their developments with age are shown in Figure 5. A downward trend in the *r*-age curves was clearly observed. Especially during the first 14 days, the hydration rate of the samples markedly declined. At the age of 14 days, the hydration rates were just about 0.15. For the sample containing less CSS, the drops of hydration rates were biggest, and the maximum drop reached about 90%. When more CSS was contained in the sample, the decline of hydration rate became slower. This phenomenon indicated that a slower hydration action of CSS can slow down the hydration rate of a sample with age.

After the age of 14, the hydration rate of all samples developed smoothly with age, and the fluctuation range of *r* was from 0.05 to 0.2. The difference in the *r*-age curves was small. Because the hydration of cement in the sample was almost completed, as shown in samples W1CS0, W2CS0 and W3CS0, the hydration rate of the sample depended on the slower hydration of CSS. The effect of CSS was not obvious.

### 3.5. Pore Structure 

**Figure 6:**
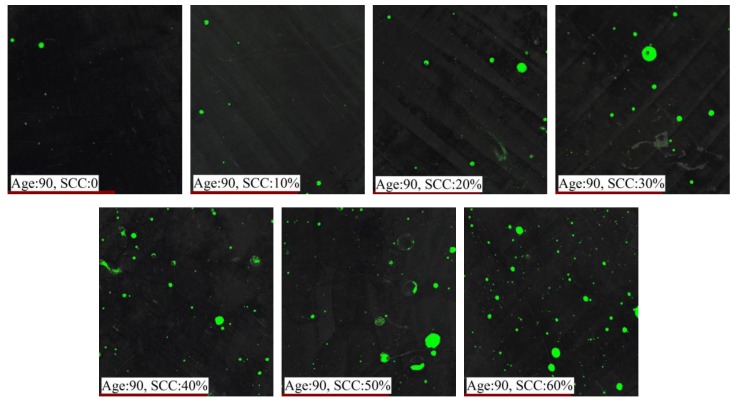
Pore structure distribution.

The pore structure distributions of the sample surfaces are shown in Figure 6. The green dots were the pores in the figures. It can be seen that there were more green dots and the size was bigger for the samples containing more CSS. This phenomenon indicated that CSS had a significant effect the pore structure of the sample. The reason for this may be the slower hydration of the sample containing CSS because β-C_2_S was the main mineral component of CSS. Thus, there were less hydration products at the same age, and there were more pores.

**Figure 7:**
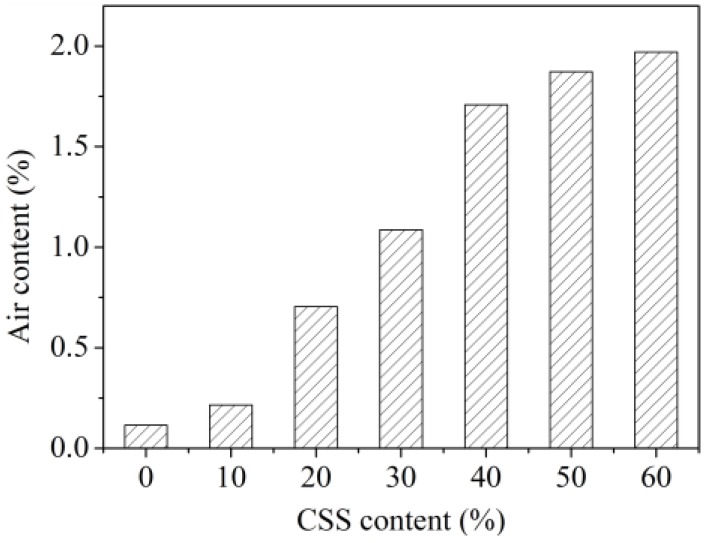
Air content in the pore.

The air content in the pores was measured and is shown in Figure 7. It can be seen that air content was higher for the samples containing more CSS. This result correlated with the amount and size of the pore. They were all due to the slower hydration of the sample containing CSS.

### 3.6. Flexural Strength

**Figure 8:**
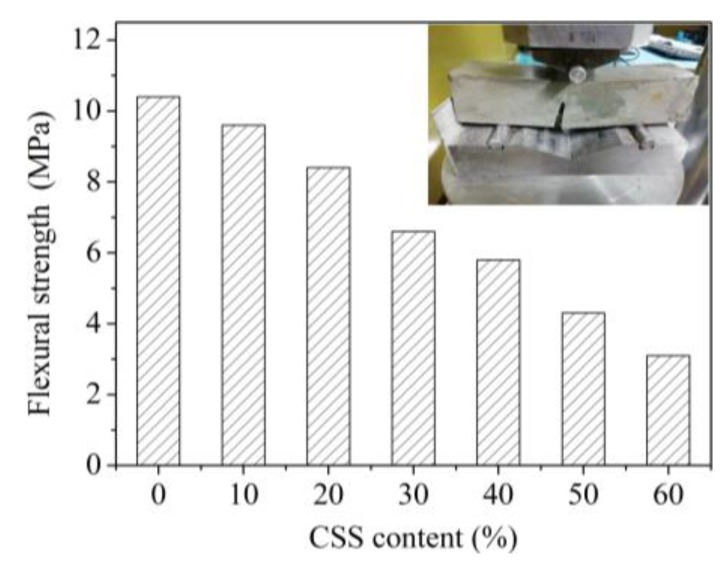
Flexural strength of the sample.

The flexural strength of the samples was tested according to Chinese standard GB/T 17671-1999 [25], and the results of this test are shown in Figure 8. It can be seen from Figure 8 that the flexural strength of the sample gradually decreased with the increase of CSS content. After comparing to the results of air content in the pore as shown in Figure 7, it was clear that the flexural strength decreased when the air content of the sample increased. The reason was the slower hydration of CSS.

Additionally, the results in the Figure 8 indicate that all samples containing CSS possessed a higher flexural strength. Even if 60% of CSS was added into the sample, the flexural strength could reach 3.1 MPa. This demonstrated that CSS was active after mechanical ball-milling.

### 3.7. SEM Analyses

**Figure 9:**
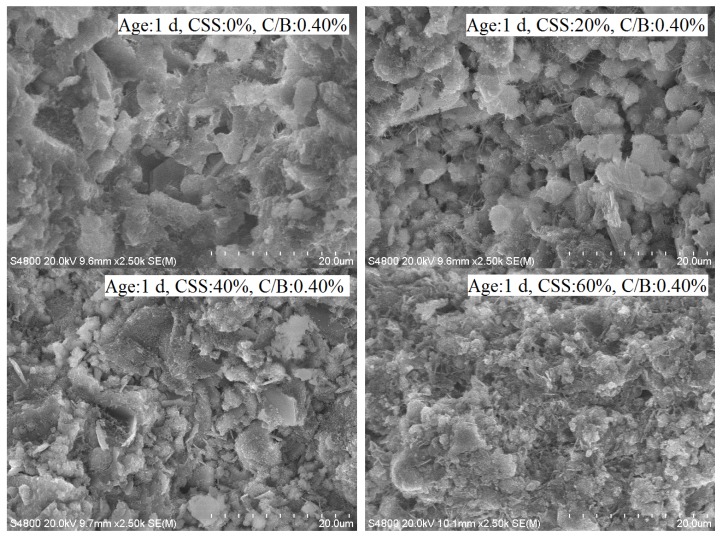

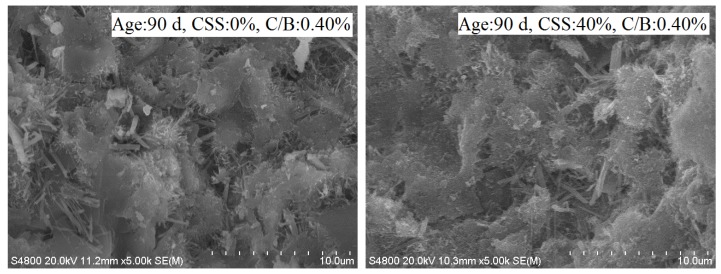
Micromorphologies of the samples.

Chemically combined water existed in the calcium silicate hydrate gel in the hydration products of the samples. The amount of calcium silicate hydrate gel determined the amount of chemically combined water. Figure 9 shows the micromorphology of the samples with different contents of CSS at different ages. The calcium silicate hydrate gel was spherical in the micromorphology. Within 14 d of age, chemically combined water mainly came from the cement in the sample, as shown in Figure 3. Therefore, the amount of spherical gel was the greatest for the sample without CSS, and the volume of the spherical gel was the greatest at the age of 1 d in Figure 9. After more CSS was added into the sample, the volume of the spherical gel decreased. The spherical gel containing a CSS content of 60% could not be found at the age of 1 d in the sample. This phenomenon in the micromorphology of the samples also showed that chemically combined water gradually decreased with an increase in CSS.

With increasing age, the hydration of the samples continued, and the amount of hydration products increased. Figure 9 shows that the amount of spherical gel increased at the age of 90 d. Many spherical gels formed links and resembled floccules. Though the hydration rate of the sample with more CSS was slow, the micromorphologies appeared very similar to the sample without CSS and with a CSS content of 40%. This indicated that the hydration degree of the sample increased as the amount of CSS increased. 

## 4. Conclusions

In the investigation, composite samples were made that contained CSS and cement. The characteristics and activity of CSS and chemically combined water, as well as the hydration degree, hydration rate, pore structure, flexural strength and micromorphology of the samples were analyzed. Some conclusions can be drawn:

(1) The main mineral composition of CSS was β-C_2_S, and its hydration was slower than that of cement.
(2) After cement was partly replaced with CSS, the hydration rates of the composite pastes containing both cement and CSS slowed down, chemically combined water could be reduced, and more aging time was needed to reach a high hydration degree.
(3) After more CSS is added into the cementitious pastes, there are more and bigger pores with higher air contents. However, flexural strength is lower.
(4) The calcium silicate hydrate gel appeared spherical in the micromorphology of cementitious paste. After more CSS was added into the sample, the volume of the spherical gel decreased, and chemically combined water decreased.
(5) Based on the results of particle size distribution, flexural strength and chemically combined water, CSS was active after mechanical ball-milling.
(6) The hydration of cementitious pastes could be regulated by addition of CSS.
(7) These results can be applied to decrease hydration heat during large concrete construction and reduce CSS discharge.

## Figures and Tables

**Table 1:** Main chemical components of raw materials.

Oxides (%)	CaO	MgO	Al_2_O_3_	Fe_2_O_3_	SiO_2_	Na_2_O	LOI
CSS	51.75	1.14	5.5	2.55	27.69	3.1	9.88
P.O 42.5 cement	55.01	2.24	7.19	2.96	23.44	0.68	2.86

**Table 2:** Paste samples containing CSS and cement.

Sample	Mix Proportion (by Mass)/%			Sample	Mix Proportion (by Mass)/%			Sample	Mix Proportion (by Mass)/%		
	CSS	Cement	W/B		CSS	Cement	W/B		CSS	Cement	W/B
W1CS0	0	100	0.35	W2CS0	0	100	0.40	W3CS0	0	100	0.45
W1CS1	10	90	0.35	W2CS1	10	90	0.40	W3CS1	10	90	0.45
W1CS2	20	80	0.35	W2CS2	20	80	0.40	W3CS2	20	80	0.45
W1CS3	30	70	0.35	W2CS3	30	70	0.40	W3CS3	30	70	0.45
W1CS4	40	60	0.35	W2CS4	40	60	0.40	W3CS4	40	60	0.45
W1CS5	50	50	0.35	W2CS5	50	50	0.40	W3CS5	50	50	0.45
W1CS6	60	40	0.35	W2CS6	60	40	0.40	W3CS6	60	40	0.45
